# Evaluating *Candidatus* Aquirickettsia rohweri gene expression upon nutrient enrichment in disease-susceptible *Acropora cervicornis*

**DOI:** 10.3389/fmicb.2026.1754183

**Published:** 2026-04-16

**Authors:** Lauren Speare, J. Grace Klinges, William C. Duke, Erinn M. Muller, Rebecca L. Vega Thurber

**Affiliations:** 1 School of Biological Sciences, Georgia Institute of Technology, Atlanta, Georgia; 2 Department of Microbiology, Oregon State University, Corvallis, OR, United States; 3 Island Conservation, Santa Cruz, CA, United States; 4 Mote Marine Laboratory, Sarasota, FL, United States; 5 Marine Science Institute, University of California at Santa Barbara, Santa Barbara, CA, United States

**Keywords:** *Acropora cervicornis*, coral disease, eutrophication, Rickettsiales, transcriptomics

## Abstract

Ocean warming, disease, and pollution have contributed to global declines in coral abundances and diversity. In the Caribbean, corals previously dominated reefs, providing an architectural framework for diverse ecological habitats, but have significantly declined due to infectious disease and anthropogenic climate change. Key species like the coral *Acropora cervicornis* are critically endangered, prompting researchers to focus on scientific endeavors to identify factors influencing coral disease resistance and resilience. We previously showed that disease susceptibility, growth rates, and bleaching risk were all associated with the abundance of a single bacterial parasite, *Candidatus* Aquirickettsia rohweri which proliferates *in vivo* under nutrient enrichment. Yet how nutrients influence parasite physiology *in vivo* remains unknown. Here, we analyzed parasite gene expression from a disease-susceptible *A. cervicornis* genotype exposed to ambient or nutrient enrichment conditions. Electron microscopy showed that *Ca.* A. rohweri was abundant in coral tissue and densely packed in mucocytes prior to nutrient enrichment. Under ambient conditions, the parasite upregulated genes involved in translation, protein maintenance, and cell envelope integrity, consistent with a conserve-and-maintain strategy. Nutrient enrichment induced expression of genes associated with central metabolism, nutrient import, stress response, host interaction, and two-component systems. Together, these results indicate that nutrient enrichment activates a growth-and-exploitation strategy, likely exacerbating parasitic pressure on *A. cervicornis*.

## Introduction

Environmental stressors such as anthropogenic-induced ocean warming, disease, and pollution have contributed to a worldwide decline in coral diversity and coverage (Huang and Roy, 2015; Hughes et al., 2017). Corals maintain intimate associations with diverse microbial symbionts; however, these intricate relationships can be disrupted by environmental disturbances, resulting in dysbiosis and coral disease (McDevitt-Irwin et al., 2017; Bourne et al., 2016; Zaneveld et al., 2016; Vega Thurber et al., 2012; Vega Thurber et al., 2014; Pawlik et al., 2016; Shaver et al., 2017). For example, the relationship between corals and their endosymbiotic dinoflagellates is dependent on oligotrophic conditions with low nitrogen availability (Wooldridge, 2010; Shantz and Burkepile, 2014). Local eutrophication has disrupted this delicate balance, resulting in increased prevalence and severity of coral bleaching and disease (Vega Thurber et al., 2014; Bruno et al., 2003; Voss and Richardson, 2006). In the Caribbean, acroporid corals that previously dominated reefs and provided the architectural framework for diverse ecological habitats have shown significant declines due to infectious disease (Williams and Miller, 2005; Kline and Vollmer, 2011; Muller et al., 2020; Patterson et al., 2002) and the effects of climate change. The staghorn coral *Acropora cervicornis*, is one of the only fast-growing taxa with branching morphologies in the region and is now considered critically endangered and functionally extinct in Florida’s coral reef (Manzello et al., 2025). This has prompted researchers to focus restoration efforts on understanding factors that promote disease susceptibility and resistance.

Recent evidence suggests that host genotype and microbiome composition significantly impact *A. cervicornis* disease susceptibility (Klinges et al., 2020; Muller et al., 2018; Drury et al., 2017; Chu and Vollmer, 2016). Resistant hosts may better tolerate potential pathogens, prevent opportunists from acting antagonistically, or house beneficial symbionts that increase host disease resistance (Chu and Vollmer, 2016). In contrast, susceptible genotypes may more easily succumb to microbial antagonism or harbor parasites that exacerbate environmental stressors. For example, *A. cervicornis* disease susceptibility was recently linked to the presence of an intracellular bacterial parasite, *Candidatus* Aquirickettsia rohweri, formerly Aquarickettsia (Klinges et al., 2020; Klinges et al., 2019; Di Lauro, 2015). *Ca.* A. rohweri abundance varies significantly with *A. cervicornis* genotype, where microbiomes of disease susceptible genotypes are dominated by *Ca.* A. rohweri (89.7%), while *Ca.* A. rohweri makes up a minor constituent of disease resistant microbiomes (2.5%) (Klinges et al., 2020; Muller et al., 2018; Klinges et al., 2023). Further, *Ca.* A. rohweri abundance was experimentally linked to reduced coral growth rates (Shaver et al., 2017) and increased infection by opportunists upon bleaching (Klinges et al., 2020).

*Ca.* A. rohweri is undergoing positive selection across the Caribbean, suggesting this taxa is highly responsive to environmental conditions (Baker et al., 2022). Ribosomal-associated genes and virulence genes, including type IV secretion system (T4SS) genes, are undergoing the greatest degree of positive selection, which is concerning given that *Ca.* A. rohweri are also transmitted horizontally between hosts (Baker et al., 2022). Like other Rickettsiales bacteria (Salje, 2021), *Ca.* A. rohweri appears to parasitize its host for nutrients, energy, and amino acids (Klinges et al., 2019). *Ca.* A. rohweri inhabit coral mucocytes, cells in the coral epidermis that produce mucus to protect against sedimentation and infection; within these specialized cells, *Ca.* A. rohweri are localized near, yet not within, endosymbiotic coral dinoflagellate cells (Klinges et al., 2019). This parasite does not encode genes to synthesize most amino acids (Klinges et al., 2019), suggesting it relies heavily on parasitism of the coral host and/or endosymbiotic dinoflagellates (Klinges et al., 2020) for resources.

Given the pervasiveness of *Ca.* A. rohweri in Caribbean acroporids and demonstrated negative effects of local nutrient pollution on coral health, significant efforts have gone toward understanding the role of nutrient enrichment on *Ca.* A. rohweri abundance (Shaver et al., 2017; Klinges et al., 2022). We previously performed a manipulative tank experiment to disentangle the individual and combined impacts of nutrient enrichment on *Ca.* A. rohweri abundance within *A. cervicornis* microbiomes (Klinges et al., 2023; Klinges et al., 2022). We exposed a disease susceptible *A. cervicornis* genotype (ML-50) and a disease resistant *A. cervicornis* genotype (ML-7) (Muller et al., 2018; Klinges et al., 2020), to elevated levels of nitrate, ammonium, phosphate, or a combination of the three for 6 weeks and evaluated microbiome composition and host fitness. Analysis of community dynamics revealed that *Ca.* A. rohweri increased in absolute abundance in response to nutrient enrichment in both host genotypes, however it remained at low relative abundances in the disease-resistant genotype, ML-7 (<0.5% of the total bacterial community) (Klinges et al., 2023). In the disease-susceptible genotype, ML-50, *Ca.* A. rohweri dominated the bacterial microbiome in all treatments and increased in both relative and absolute abundance, while overall microbiome diversity declined in response to nutrient enrichment (Klinges et al., 2022). Genotype ML-50 corals showed an increase in visual dinoflagellate symbiont density yet a decrease in coral growth in response to elevated nutrients, suggesting that nutrient enrichment promotes coral microbiome dysbiosis and reduced coral fitness (Klinges et al., 2022).

Given that nutrient enrichment increased *Ca.* A. rohweri abundance and dominance within *A. cervicornis* genotype ML-50, this study investigates how nutrient enrichment impacts *Ca.* A. rohweri activity by analyzing *Ca.* A. rohweri gene expression data from samples in a “no amendment” treatment versus a “nutrient enrichment” treatment. Here, we describe the localization of *Ca.* A. rohweri within coral tissue and show evidence consistent with the predicted role of *Ca.* A. rohweri as a nutrient-responsive *A. cervicornis* parasite.

## Methods

### Experimental design and sample collection

To test the impacts of nutrient enrichment on *A. cervicornis* health and *Ca.* A. rhoweri activity, a tank experiment was conducted as previously described (Klinges et al., 2022; Klinges et al., 2023) at the Mote Marine Laboratory International Center for Coral Reef Research and Restoration (24°39′41.9”N, 81°27′15.5”W) in Summerland Key, Florida from April to June 2019. The experiment was conducted in 4.7 L flow-through, temperature-controlled tanks with natural locally-sourced sea water from the Atlantic side of the Keys. Sand- and particle-filtered water was fed from header tanks to tanks by powerheads fitted with tubing splitters at a flow rate of 256.66 ± 43.89 mL per minute (approximately 1 h for full water turn over). Tanks were located outdoors under natural light regimes with the addition of 75% shade cloth to account for shallow tank depth. Tanks were divided between two flow-through seawater raceways, which allowed for temperature regulation of individual Tanks. Water temperatures were maintained at an average of 27.19 ± 0.6 °C by a boiler and chiller using a dual heat exchanger system connected to header tanks and individual raceways. Header tank pH was stabilized at ~8.0 by aeration and mixed via a venturi pump system. Tanks were cleaned every third day to prevent overgrowth of coral fragments with diatoms or algae.

Fragments (~5 cm) of two *Acropora cervicornis* genotypes (Coral Sample Registry accession: fa13971c-ea34-459e-2f13-7bfddbafd327), ML-50 a highly disease-susceptible genet, and ML-7, a disease-resistant genet (Muller et al., 2018; Klinges et al., 2019), were collected from the Mote Marine Laboratory *in situ* coral nursery in Looe Key in April 2019. Corals were randomly assigned treatments, placed into one of three replicate tanks assigned to each treatment (Supplementary Table S2; Klinges et al., 2022); nutrient treatment tanks were randomly distributed throughout each raceway, with each raceway containing at least one tank from each treatment group. Corals in the no amendment treatment and elevated nutrient treatments were analyzed in this study. The nutrient enrichment treatment was treated with Osmocote, a controlled release fertilizer containing nitrate (NaNO_3_), ammonium (NH_4_Cl), and phosphate (Na_3_PO_4_), four times a day for 42 days (6 weeks). After each nutrient addition, flow in tanks was stopped for 1 h to create an hour-long nutrient pulse. This was subsequently followed by 1 h of dilution and 4 hrs of exposure to ambient conditions before the next enrichment event. After initial acclimation three samples were collected from each coral genotype (representing a baseline for the ambient treatment); after 6 weeks of treatment exposure, corals were collected at random from the no amendment treatment and nutrient enrichment treatment; replicate fragments from a given treatment were collected from unique tanks (*n* = 18 coral fragments total). Using sterile bone cutters, tissue was scraped from each fragment (avoiding the apical tip) and added directly to 2 mL tubes containing 0.5 mL DNA/RNA shield (Zymo Research) and Lysing Matrix A (MP Biomedicals, 0.5 g garnet matrix and one 1/4″ ceramic sphere). Tubes were immediately preserved at −80 °C until further processing. Total RNA was extracted from 500 μL of tissue slurry using the E. Z. N. A.® DNA/RNA Isolation Kit (Omega Bio-Tek) and then stored at −80 °C until further processing.

### Electron microscopy

Samples were processed for Electron Microscopy at Oregon State University. Samples were decalcified for 5 weeks with a 10% EDTA (pH 7) solution; the solution was replaced three to four times each week. After the skeleton was fully dissolved, the remaining tissue was fixed with Karvosky fixative (2% paraformaldehyde, 2.5% glutaraldehyde, 0.1 M buffer) overnight. Samples were then embedded in agar for post-fixation staining performed by Teresa Sawyer at the Oregon State University Electron Microscope Facility. Briefly, coral tissue was first rinsed with 0.1 M sodium cacodylate buffer. Post fixation was conducted in 1.5% potassium ferrocyanide and 2% osmium tetroxide in deionized water. Samples then underwent T-O-T-O staining, uranuyl acetate, and lead aspartate fixation. Samples were sequentially dehydrated in a range of increasing concentration acetone mixtures for 10–15 min: 10, 30, 50, 70, 90, 100, 100%. Finally, samples were infiltrated with Araldite resin and ultrathin sectioned. Images were collected on a FEI Helios Nanolab 650 at the Oregon State University Electron Microscopy Facility.

### Sequencing and bioinformatics analysis

Residual DNA contamination was removed from RNA isolates using the RQ1 RNase-Free DNase (Promega). Ribosomal RNA was removed using equal parts ‘plant leaf’, ‘human/mouse’ and ‘bacteria’ Ribo-Zero kits (Illumina). RNA quality and concentration were verified by BioAnalyzer (Agilent Technologies, Santa Clara, CA) and quantitative PCR, respectively. cDNA library prep and sequencing were performed at Oregon State University’s Center for Quantitative Life Sciences (CQLS) Core Laboratories with the HiSeq 3,000 platform. 18 coral fragments, representing three biological replicates for each genotype per treatment per time point, were sequenced (*n* = 18). Quality scores were calculated for each sequence using FastQC and MultiQC (version 0.12.1) (Ewels et al., 2016; Supplementary Table S3); low-quality scores (average score <20 across 5 bp) were removed. Adapters were trimmed using bbduck (BBTools User Guide); successful trimming was confirmed using FastQC/MultiQC (Ewels et al., 2016). Forward and reverse reads were then interleaved using reformat (BBTools User Guide), mapped to the *Ca.* A. rohweri genome (Klinges et al., 2019) using BowTie2 (version 2.5.1) (Langmead and Salzberg, 2012), and counted using HTSeq-count (version 2.0.3) (Putri et al., 2022). To evaluate potential coral host or symbiont cross-mapping, quality-filtered reads from each sample were aligned independently to the *A. cervicornis* or *Symbiodinium fitti* genome (Reich et al., 2021) genome using Bowtie2 with the –very-sensitive preset. Mapped read IDs were extracted from host and *Ca.* A. rohweri alignments, retaining only high-confidence mappings (MAPQ ≥ 20) and excluding secondary or unmapped reads. The sets of mapped read IDs were then compared between host/algal symbiont and bacterial alignments for each sample. No overlapping read IDs were detected between host-mapped and *Aquirickettsia*-mapped reads, or *S. fitti*-mapped and *Ca.* A. rohweri reads (Supplementary Table S4) indicating that *Ca.* A. rohweri alignments were not attributable to coral host or symbiont cross-mapping. The limit of detection for each gene was one read per gene. Genes that had detectable expression in three or fewer samples were not included in this analysis. We did not detect genes with transcripts is only one treatment (ie. Gene A was expressed in all no amendment samples and not in any nutrient enrichment samples).

Although 18 libraries were initially sequenced, *Ca.* A. rohweri transcripts were rare in the disease-resistant genet, ML-7 (0.003% of the ML-7 metatranscriptome, or roughly 1,000 out of ~37 million reads), precluding robust comparisons of *in situ Ca.* A. rohweri activity between disease susceptible and resistant hosts. Additionally, because the “ambient” tank conditions were nutrient enriched relative to the offshore nursery where corals were collected, these samples did not accurately reflect baseline conditions and were not directly comparable to samples collected 6 weeks after experimental exposure. Thus, all downstream analyses (including all tables and figures) were restricted to ML-50 genotype samples collected 6 weeks after exposure to ambient tank conditions or nutrient enrichment (*n* = 6).

The vegan package in R was used to perform principal coordinates analysis (PCoA) using the Bray–Curtis dissimilarity index (Dixon, 2003), PERMANOVA using the Adonis function (Anderson, 2017), and beta-diversity using the permutest.betadisper function (Dixon, 2003). A power analysis for PERMANOVA was performed using the MASS and vegan packages in R on a simulated dataset with increasing numbers of samples per group (from 3 to 12) and plotted using a power curve. Gene categorization was performed based on Kyoto Encyclopedia of Genes and Genomes Orthology Pathway designations (Kanehisa and Goto, 2000). Differential gene expression (DGE) analysis was performed through EdgeR Bioconductor package (version 3.36.0) (Robinson et al., 2010), which uses a trimmed mean of M-values (TMM) normalization method and graphed via volcano plots (Supplementary Table S5). Hierarchical clustering analyses were performed using the ggplot heatmap function in R and include TMM normalization. For heatmap visualization TMM-normalized transcript counts were scaled across samples for each gene (z-score transformation), where “low” versus “high” expression reflects relative expression within a gene rather than absolute transcript abundance. Data were graphed in Graphpad Prism, or R and edited for publication using Inkscape 1.0.^1^

### Phylogenetic analyses

Multilocus two-component system (TCS) phylogenetic analyses were performed using the response regulator and histidine kinase for the three two-component systems encoded by *Ca.* A. rohweri: NtrY-NtrX, PhoR-PhoB, and EnvZ-OmpR. Published sequence data from the genomes of Rickettsiales bacteria were collected into separate TCS files and combined into a single concatenated sequence for each TCS (ordered histidine kinase, response regulator). Concatenated sequences were aligned (ClustalW) (Larkin et al., 2007) and phylogenetic reconstructions assuming a tree-like topology were created with MEGAX via maximum likelihood (ML) (Stecher et al., 2020). Gaps were treated as missing. The LG model with non-uniformity of evolutionary rates among sites may be modeled by using a discrete Gamma distribution (+G) with 5 rate categories and by assuming that a certain fraction of sites are evolutionary invariable (+I) was the most optimal evolutionary model. Tree inference was applied heuristically via the nearest-neighbor-interchange [NNI] method without a branch swap filter for 1,000 bootstrap replications. Congruence among distance matrices (CADM) analysis was performed to determine phylogenetic congruence (Campbell et al., 2011). Phylogenetic trees were visualized with MEGAX and edited for publication with Inkscape 1.0.^2^

### Availability of data and materials

Sequences were submitted to the NCBI SRA database under BioProject PRJNA1048415. Code for this work can be found at https://github.com/spearel/Acer-RICA-Nutrients-Mote2019.

## Results and discussion

### *Candidatus* Aquirickettsia rohweri is prevalent in *Acropora cervicornis* genotype ML-50 mucocytes and tissue

**Figure 1:**
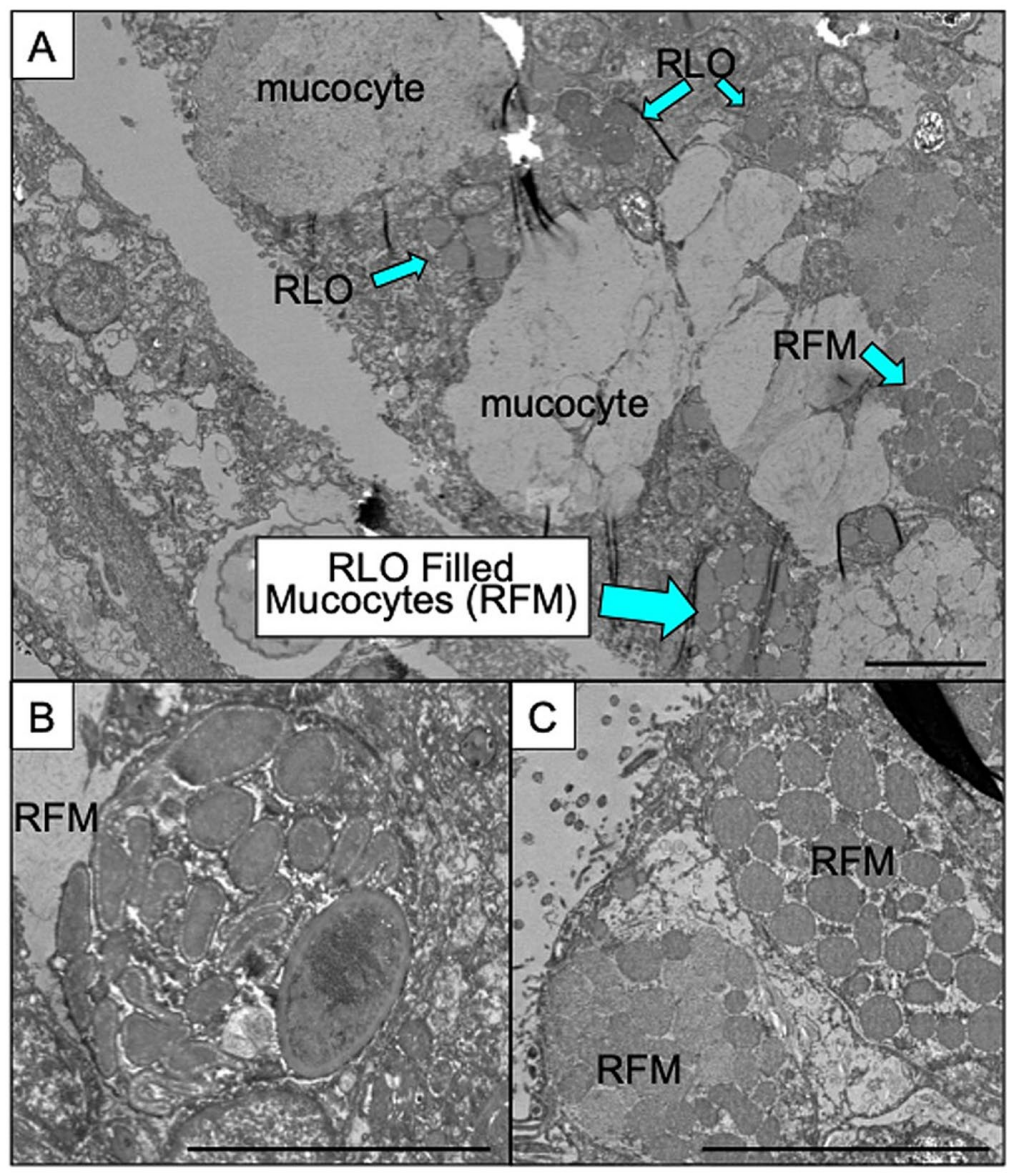
Representative images of *Acropora cervicornis* tissues during experiment. **(A)** Examples of apparently normal mucocytes and mucocytes filled with Rickettsiales like organisms (RLOs) as well as clusters of RLOs outside of mucocytes. **(B)** Individual RLO filled mucocyte (RFM) with multiple bacterial cells. **(C)** Two side-by-side mucocytes filled with bacterial cells at the edge of the epithelium ready to release infected mucocyte into the environment. Scale bars in all images indicate 5 μm.

To provide context for our gene expression data, we first examined the spatial localization of intracellular bacteria within *A. cervicornis* ML-50 tissue via transmission electron microscopy (Figure 1). We observed abundant intracellular bacteria with morphology consistent with Rickettsiales-like organisms (RLOs) (Klinges et al., 2019), located both outside of coral mucocytes and also densely packed within mucocytes within the gastrodermal cells and epidermis (Figure 1). Cells found inside these mucocytes were ~1–2.5 μm in length and 0.5 μm in width. While untrastructural features alone do not permit definitive taxonomic identification, these observations are consistent with prior microbiome surveys of ML-50 in which *Ca.* A. rohweri dominates the microbiome (Klinges et al., 2022). Based on this concordance, we can infer that these cells are consistent with *Ca.* A. rohweri. The presence of these densely packaged mucocytes positioned near the cell surface could suggest that bacteria-filled mucocytes could represent a potential route of horizontal transmission (Baker et al., 2022) either between hosts or into the surrounding water column.

### Nutrient enrichment shifts *Candidatus* Aquirickettsia rohweri gene expression

To examine how nutrient enrichment affects *Ca.* A. rohweri activity *in vivo*, we analyzed *Ca.* A. rohweri gene expression within the disease susceptible *A. cervicornis* genet, ML-50, that were maintained in tanks or exposed to elevated nutrients (Osmocote), approximately 5-7x times nutrient conditions (Supplementary Table S1; Klinges et al., 2022). Tank source water had elevated levels of dissolved nitrogen compared to Mote’s *in situ* Looe Key nursery, where corals were raised (~3.7x source water; Supplementary Table S1). Thus, all corals experienced a moderate increase in nitrogen (nitrate-nitrite) relative to their collection environment, and “ambient” tank conditions should not be interpretated as a true oligotrophic reference.

Transcripts that mapped to the *Ca.* A. rohweri genome made up approximately 0.09% of the entire ML-50 metatranscriptome for each sample, or roughly 31,000 transcripts out of ~35 million paired-end fragments in each sample (Supplementary Figure S1; Supplementary Table S4). We detected transcripts for 69% of the coding region of the *Ca.* A. rohweri genome across all samples and treatments (Supplementary Figure S1C). This level of coverage is substantial considering *Ca.* A. rohweri is a single bacterium within the complex coral holobiont; in most other studies reads for the entire bacterial component of coral metatranscriptomes typically account for less than 0.02% of all transcripts (Daniels et al., 2015; Li et al., 2023). Genes with transcripts in fewer than four samples were removed; thus, genes with naturally low levels of transcription may not be included in this analysis.

**Figure 2:**
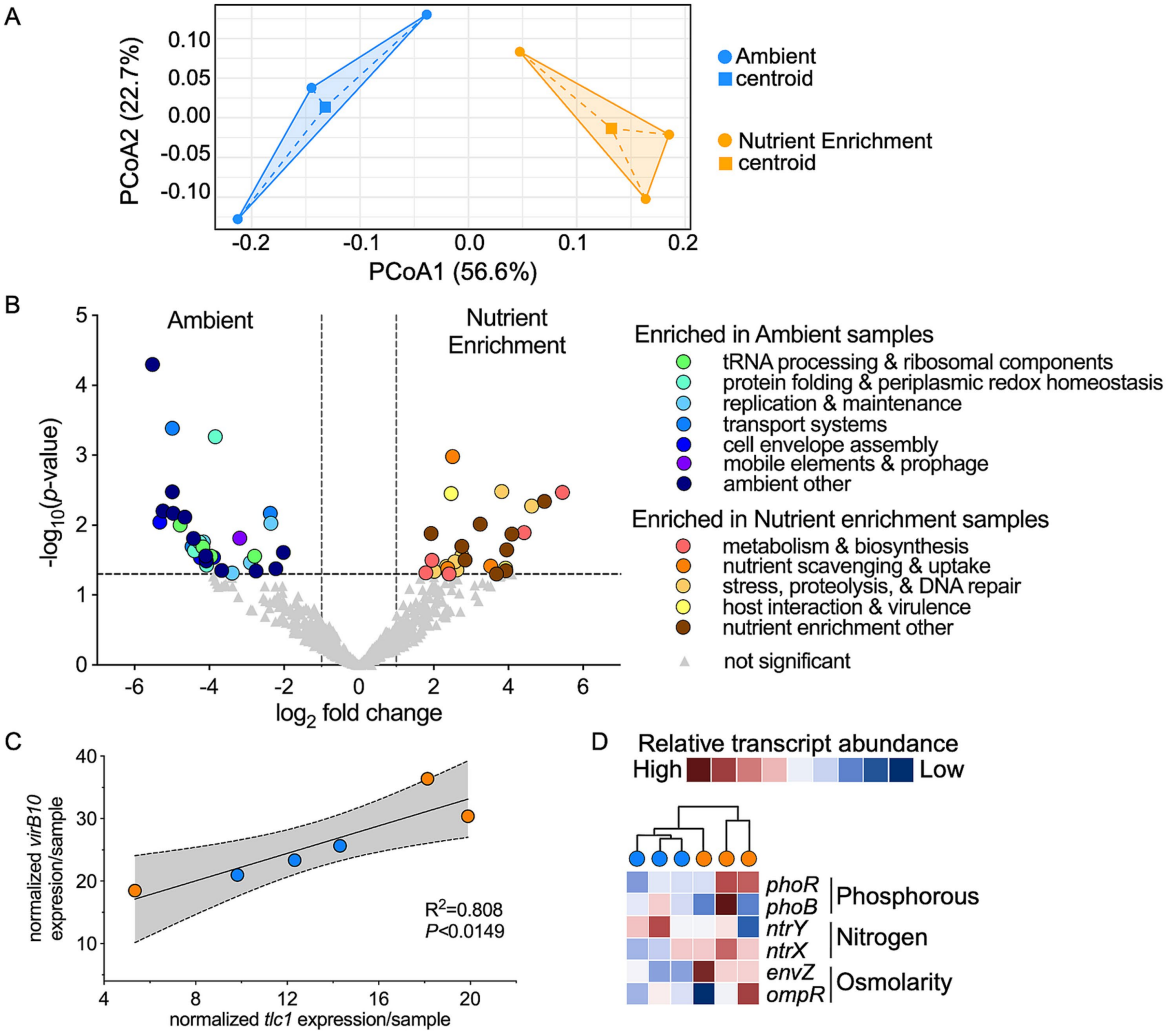
*Ca. A. rohweri* gene expression is shaped by nutrient enrichment. **(A)** Principal coordinates analysis (PCoA) based on Bray-Curtis dissimilarities of reads by nutrient treatment, indicated by symbol color: ambient (blue), nutrient enrichment (orange). Percentages on each axis indicate the amount of variation explained by each axis. Centroids are shown as squares. Results of PERMANOVA test were not significant (*p* < 0.10). **(B)** Volcano plots showing pairwise comparative analysis of transcript abundance between ambient and nutrient enriched samples. Light gray triangles were not significantly differentially expressed; all other symbols indicate genes that were significantly differentially expressed: a magnitude fold change > |1| log_2_ (vertical dashed lines on *x*-axis) and adjusted *p*-value <0.05 horizontal dashed line on *y*-axis. **(C)** Linear regression analysis between normalized *tlc1* and *virB10* expression. Gray area indicates 95% confidence intervals. **(D)** Hierarchical clustering analysis and heatmaps displaying relative transcript abundance for two-component system genes. Each circle represents a sample and color indicates treatment: ambient (blue), nutrient enrichment (orange). Transcript abundance was TMM-normalized and scaled across samples for each gene (*z*-score transformation); colors represent relative expression within a gene (red = higher relative expression, blue = lower relative expression) rather than absolute transcript abundance.

To begin understanding the impact of nutrient enrichment on *Ca.* A. rohweri activity in disease-susceptible *A. cervicornis* tissue, we first performed a principal coordinate analysis (PCoA) based on Bray-Curtis dissimilarities on *Ca.* A. rohweri transcripts. PCoA showed distinct clustering by treatment, with treatment centroids separated along PCoA1 axis (56.6% of variation) (Figure 2A). PERMANOVA revealed a non-significant treatment effect (*R*^2^ = 0.47, *p* = 0.10), with homogeneous dispersion confirmed by betadisper (*p* = 0.8772). While these data suggest a potential biological response of *Ca.* A. rohweri to nutrient enrichment, the current sample size is insufficient to test this hypothesis with statistical rigor using PERMANOVA. Power simulations suggest that four samples per treatment would achieve >80% power (Supplementary Figure S2), suggesting that the non-significant PERMANOVA result is likely due to low sample size rather than absence of a biological effect.

### *Candidatus* Aquirickettsia rohweri expression exists along spectrum depending on ambient nutrient conditions

To better understand the transcripts driving the differences between treatments, we generated a volcano plot to identify differentially expressed transcripts. EdgeR analysis revealed 77 genes were significantly differentially expressed, where 34 were upregulated in ambient samples, indicating downregulation under nutrient enrichment (Figure 2B; Supplementary Table S5). Under ambient conditions, *Ca.* A. rohweri upregulated a suite of genes associated with translation, replication, and envelope-assembly factors. We observed upregulation of genes encoding tRNA processing and ribosomal components (alanine-tRNA ligase, TruA, TrmD, and ribosomal protein S10), protein folding and periplasmic redox homeostasis (DnaJ chaperone, signal peptidase II, and TlpA redox/disulfide isomerase), baseline replication and maintenance factors (FtsA, dUTP diphosphatase, and DNA replication factor DciA) and cell envelope assembly components (lipid A disaccharide synthase, UDP-N-acetylglucosamine transferase, and LptF/G [LPS export]). These expression patterns suggest general protein maintenance and synthesis, baseline cell replication, and investment in maintaining cell envelope integrity. Other transcripts with elevated expression in ambient samples encoded transport systems that mediate nutrient uptake and efflux toxic ions (ExbD – TonB system, MFS transporter, and fluoride efflux transporter) and mobile elements and prophage genes (major capsid phage protein, IS66 transposase) (Figure 2B), indicating possible phage/insertion sequence activity or prophage expression. Taken together, these data suggest that under ambient conditions, *Ca.* A. rohweri expresses machinery for accurate protein production, envelope maintenance, and controlled cell-cycle activity, consistent with a “conserve and maintain” expression profile.

To evaluate how nutrient enrichment shifts *Ca.* A. rohweri expression, we examined the 33 genes with significantly higher expression in nutrient enriched samples compared to ambient (Figure 2B; Supplementary Table S5). These transcripts fell under four categories: (1) metabolism and biosynthesis, (2) nutrient scavenging and uptake, (3) stress, proteolysis, and DNA repair, and (4) host interaction and virulence. Higher expression of citrate synthase, class II aldolase, 3-oxoacyl reductase, glycine-tRNA ligase *β*, and peptide deformylase suggest high carbon metabolism, replication, and growth in nutrient enrichment. This expression is consistent with the increased *Ca.* A. rohweri abundance observed in our experimental data (Klinges et al., 2022). *Ca.* A. rohweri upregulated expression of several transporters (dicarboxylate/citrate transporter SlC13, ferrous iron transporter B, and peptidoglycan recycling transporter AmpG) suggesting increased import of host-derived compounds. Upregulated stress, proteolysis, and DNA repair transcripts (thioredoxin, flavodoxin, HslV protease, exodeoxyribonuclease III, and an EVE-domain containing protein) indicate higher metabolic activity associated with reactive oxygen species, protein and DNA damage consistent with rapid cell growth (Jaramillo-Riveri et al., 2022). Upregulated host interaction and virulence expression included genes encoding a type IV secretion system (T4SS) protein (VirB9), cationic antimicrobial peptide (CAMP) resistance, a flagellar activator (FlrC), and a toxin/antitoxin Fic family protein. VirB9 is a T4SS outer-membrane surface subunit (Banta et al., 2011), therefore elevated expression of *virB9* may reflect increased host-contact structures. FlrC is a transcriptional activator of flagellar biogenesis and motility (Correa et al., 2000). Although *Ca.* A. rohweri is transmitted horizontally between hosts (Baker et al., 2022) and expresses its flagellar genes at a relatively high level within our dataset, flagellar expression does not correlate with nutrient enrichment (Supplementary Figure S3A), nor does *flrC* expression correlate with *fliC* (flagellin), or other motility gene expression (Supplementary Figure S3). Typically, flagellated bacteria have similar *flrC, fliC,* and other motility gene expression profiles (Syed et al., 2009). Thus, FlrC may have been repurposed in *Ca.* A. rohweri. Overall, these expression patterns suggests that nutrient enrichment, which induces host stress (Klinges et al., 2022), triggers a growth and exploitation strategy, whereby *Ca.* A. rohweri ramps up central metabolism and invests in host interaction machinery. Thus, like other intracellular bacteria (Drew et al., 2021; Salje, 2021), *Ca.* A. rohweri exists along a continuum from a persistence-oriented metabolic state to highly interactive, exploitative lifestyle in response to environmental conditions and host state.

We hypothesize that host genotype may shape where *Ca.* A. rohweri falls along this persistence-exploitation continuum. Genotype-level differences in microbiome structure, particularly *Ca.* A. rohweri dominance (Epstein et al., 2025), has been strongly linked to disease susceptibility (Klinges et al., 2020). Resistant genotypes may constrain parasite growth or exploitative activity by altering intracellular conditions, maintaing tighter immune regulation, or fostering microbial symbionts that suppress parasitism, thereby biasing *Ca.* A. rohweri toward a lower-activity, persistence-orientated state regardless of ambient nutrient conditions. Regardless of whether this parasite exhibits similar persistence-exploitation continuum patterns across host genotypes, reduced abundance alone may substantially limit its impact on host physiology in disease resistant genotypes.

### *tlc1* expression correlates with T4SS gene expression

Given the high expression of *virB9* in nutrient enriched samples, and the energy requirements for assembling and firing complex secretion systems, we investigated expression of the entire T4SS and *tlc1. Ca.* A. rohweri cannot synthesize its own ATP and rather siphons ATP from host cells, using the MAMP *tlc1* which encodes an ATP/ADP antiporter (Klinges et al., 2019). We observed significantly higher expression of *virB9*, which was detected in our EdgeR analysis (Figure 2B), yet no other T4SS genes nor *tlc1* had significantly higher expression in nutrient enriched compared to ambient samples (Supplementary Figure S4). A possible explanation for the observed *tlc1* expression is that *tlc1* is constitutively expressed, which would ensure continuous and immediate ATP import across host metabolic states. It is also possible that *tlc1* is regulated at the post-transcriptional or post-translational level. We did, however, detect a strong positive correlation between expression of *tlc1* and *virB10* (Figure 2C, 80.8% of variation), suggesting that ATP import and assembly or competence of the T4SS are linked. VirB10 is a structural and regulatory component of many *rvh* T4SSs that senses bacterial intracellular ATP levels to coordinate protein translocation (Banta et al., 2011). A strong *tlc1 virB10* correlation suggests that *Ca.* A. rohweri requires imported ATP to energize T4SS function.

### Two component system expression is linked with nutrient enrichment

Two-component systems (TCSs) allow bacteria to sense changes in environmental stimuli and mediate an adaptive response, mainly through changes in gene expression, and TCSs frequently regulate virulence factors of pathogenic bacteria (Beier and Gross, 2006). *Ca.* A. rohweri encodes three two-component systems to sense and respond to phosphorus (PhoR-PhoB), nitrogen (NtrY-NtrX), and osmolarity changes (EnvZ-OmpR). The three TCSs encoded by *Ca.* A. rohweri were significantly upregulated under nutrient enriched compared to ambient samples (Figure 2D, two-way ANOVA with Tukey’s multiple comparison test (*p <* 0.03)). This observation suggests that like the majority of two-component systems described (Bijlsma and Groisman, 2003), these TCSs are controlled via positive feedback in response to nutrient enrichment. Given that *Ca.* A. rohweri lacks complete nitrogen metabolism pathways (Klinges et al., 2019), elevated nitrogen sensed by the NtrY-X system may serve as a signal for elevated amino acid and/or sugar production by endosymbiotic dinoflagellates. Nitrogen enrichment is known to promote dinoflagellate symbiont proliferation (De’ath and Fabricius, 2010; Lapointe and Bedford, 2011; Fabricius et al., 2005; D’Angelo and Wiedenmann, 2014) while phosphate is thought to have a lesser impact given that its availability is controlled by the coral host via active transport (Godinot et al., 2009; Jackson et al., 1989). Therefore, dedicating the NtrY-X system as a sensor of dinoflagellate symbiont activity would prepare *Ca.* A. rohweri to quickly siphon photosynthates from the host or other members of the microbiome. Paired experimental data from our previous study, however, indicated that inorganic phosphorus rather than nitrogen, was the primary nutrient driving shifts in *Aquirickettsia* abundance (Klinges et al., 2022).

Given the limited experimental evidence determining the function of these two-component systems in Rickettsiales, we sought to assess the evolutionary relationships among them and Rickettsiales phylogeny. We constructed a series of maximum-likelihood phylogenetic trees, as described in (Speare et al., 2021), using histidine kinase and response regulator amino acid sequences for each two-component system. All three two-component systems were phylogenetically congruent to one another, suggesting a shared evolutionary history (Supplementary Figure S5). To determine the evolutionary relationship of these two-component systems to Rickettsiales phylogeny, we constructed a consensus phylogenetic tree of all three two component-systems and compared it to a 16S rRNA maximum likelihood tree. Congruence among distance matrices (CADM) analysis revealed that there was phylogenetic congruence between two-component systems and 16S (Supplementary Figure S6). The high conservation of these two-component systems to one another and strain phylogeny supports the distinct, yet important function of each of these systems for *Ca.* A. rohweri fitness within host tissue.

## Conclusion

Our results demonstrate that nutrient enrichment contributes to a shift in *Ca.* A. rohweri gene expression from a maintenance-oriented profile under ambient tank conditions to a growth- and exploitation-focused state under nutrient enrichment. Elevated nutrients stimulated expression of metabolic, stress-response, transport, and host-interaction genes including components of the *rvh* T4SS and all three two-component systems. Our observations align with paired experimental data showing increases in *Ca.* A. rohweri abundance and suggest that nutrient enrichment likely enhances this parasite’s capacity to acquire host-derived resources and interact with host cells. Together, our findings indicate that nutrient enrichment intensifies parasitic pressure on *A. cervicornis*, potentially contributing to declines in host health, resilience, and disease resistance.

## Data Availability

The datasets presented in this study can be found in online repositories. The names of the repository/repositories and accession number(s) can be found at: https://www.ncbi.nlm.nih.gov/, PRJNA1048415.

## Footnotes

1
http://inkscape.org/

2
http://inkscape.org/
