# Supplementary material for: Evaluating Candidatus Aquirickettsia rohweri gene expression upon nutrient enrichment in disease-susceptible Acropora cervicornis
Source: Front Microbiol. 2026 Apr 16;17:1754183. doi: 10.3389/fmicb.2026.1754183 (PMC13128583; doi:10.3389/fmicb.2026.1754183)
Supplement: Supplementary file 1 [file Data_Sheet_1.docx]

**Supplemental Tables & Figures**.

**Table S1. Nutrient levels for the enrichment experiment.** Nutrient level was assessed by AutoAnalyzer at Mote Marine Laboratory in Sarasota Florida. Nitrogen was measured as dissolved nitrate-nitrite, and ammonia; phosphorus was measured at dissolved orthophosphate. Data are presented in micromolar concentrations.

| **Treatment** | **Nitrogen, Nitrate-Nitrite (dissolved μm)** | **Nitrogen, Ammonia (dissolved μm)** | **Phosphorous, Orthophosphate (dissolved μm)** |
| --- | --- | --- | --- |
| Looe Key Reef 2010s  (Lapointe *et al* 2019) | 0.55 +/- 0.05 | 0.57 +/- 0.04 | 0.11 +/- 0.01 |
| Ambient aquarium conditions | 2.04 +/- 0.06 | 0.81 +/- 0.13 | 0.12 +/- 0.01 |
| Nutrient enrichment treatment | 12.6 +/- 5.22 | 5.81 +/- 2.34 | 0.62 +/- 0.25 |

**Table S2. Sample-level metadata for experimental balance.** Sample ID included here represents sequences from *_R1 and *_R2 samples shown in Table S3.

| **Sample ID** | **Treatment** | **Tank replicate per treatment** | **Collection Date** |
| --- | --- | --- | --- |
| s016 | Ambient | 1 | 6/21/2019 |
| s017 | Ambient | 2 | 6/21/2019 |
| s018 | Ambient | 3 | 6/21/2019 |
| s019 | Nutrient Enrichment | 1 | 6/21/2019 |
| s020 | Nutrient Enrichment | 2 | 6/21/2019 |
| s021 | Nutrient Enrichment | 3 | 6/21/2019 |

**Table S3. Sequence quality scores for raw sequences (initial fastQC) and trimmed sequences**. R1 indicates transcripts in the forward direction and R2 indicates transcripts in the reverse direction.

| **Sample ID** | **Initial-fastQC** | | | | **Trimmed-reads-fastQC** | | | |
| --- | --- | --- | --- | --- | --- | --- | --- | --- |
|  | **total sequences** | **poor quality** | **length** | **%GC** | **total sequences** | **poor quality** | **length** | **%GC** |
| s016_R1 | 34069573 | 0 | 151 | 42 | 34069573 | 0 | 40-151 | 41 |
| s016_R2 | 34069573 | 0 | 151 | 41 | 34069573 | 0 | 40-151 | 41 |
| s017_R1 | 35074047 | 0 | 151 | 43 | 35074047 | 0 | 40-151 | 42 |
| s017_R2 | 35074047 | 0 | 151 | 43 | 35074047 | 0 | 40-151 | 42 |
| s018_R1 | 40292890 | 0 | 151 | 45 | 40292890 | 0 | 40-151 | 44 |
| s018_R2 | 40292890 | 0 | 151 | 44 | 40292890 | 0 | 40-151 | 44 |
| s019_R1 | 34788394 | 0 | 151 | 46 | 34788394 | 0 | 40-151 | 45 |
| s019_R2 | 34788394 | 0 | 151 | 46 | 34788394 | 0 | 40-151 | 45 |
| s020_R1 | 34526289 | 0 | 151 | 43 | 34526289 | 0 | 40-151 | 41 |
| s020_R2 | 34526289 | 0 | 151 | 42 | 34526289 | 0 | 40-151 | 41 |
| s021_R1 | 32727004 | 0 | 151 | 42 | 32727004 | 0 | 40-151 | 41 |
| s021_R2 | 32727004 | 0 | 151 | 42 | 32727004 | 0 | 40-151 | 41 |

**Table S4. Mapping reporting for processed transcripts.** Data are reported as total counts of transcripts mapped to the *Candidatus* Aquirickettsia rohweri, *Symbiodinium fitti*, or *Acropora cervicornis* genome. Results of a cross-mapping sensitivity check are reported in the final four columns. No overlapping reads were detected across samples.

| **Sample ID** | ***Ca.* A. rohweri** | ***S. fitti*** | ***A. cervicornis*** | **% of *Ca.* A. rohweri reads mapped to *S. fitti*** | **% of *S. fitti* reads mapped to *Ca.* A. rohweri** | **% of *Ca.* A. rohweri reads mapped to *A. cervicornis*** | **% of *A. cervicornis* reads mapped to *Ca.* A. rohweri** |
| --- | --- | --- | --- | --- | --- | --- | --- |
| s016 | 26,452 | 671,273 | 7,200,167 | 0 | 0 | 0 | 0 |
| s017 | 33,547 | 1,117,001 | 9,496,599 | 0 | 0 | 0 | 0 |
| s018 | 30,816 | 568,487 | 13,937,119 | 0 | 0 | 0 | 0 |
| s019 | 19,861 | 776,277 | 6,947,676 | 0 | 0 | 0 | 0 |
| s020 | 41,089 | 1,214,362 | 4,731,318 | 0 | 0 | 0 | 0 |
| s021 | 36,191 | 881,571 | 4,534,970 | 0 | 0 | 0 | 0 |

**( please see Excel file ‘Table 5’ ) Table S5. Results of edgeR differential gene expression analysis.** Data are presented as geneID (column A), KO designation (column B) where “0” indicates no KO designation was assigned by KEGG Orthology annotation, log2 Fold Change (column C), and adjusted p-values arranged according to K0 designation and display within figure 2B.


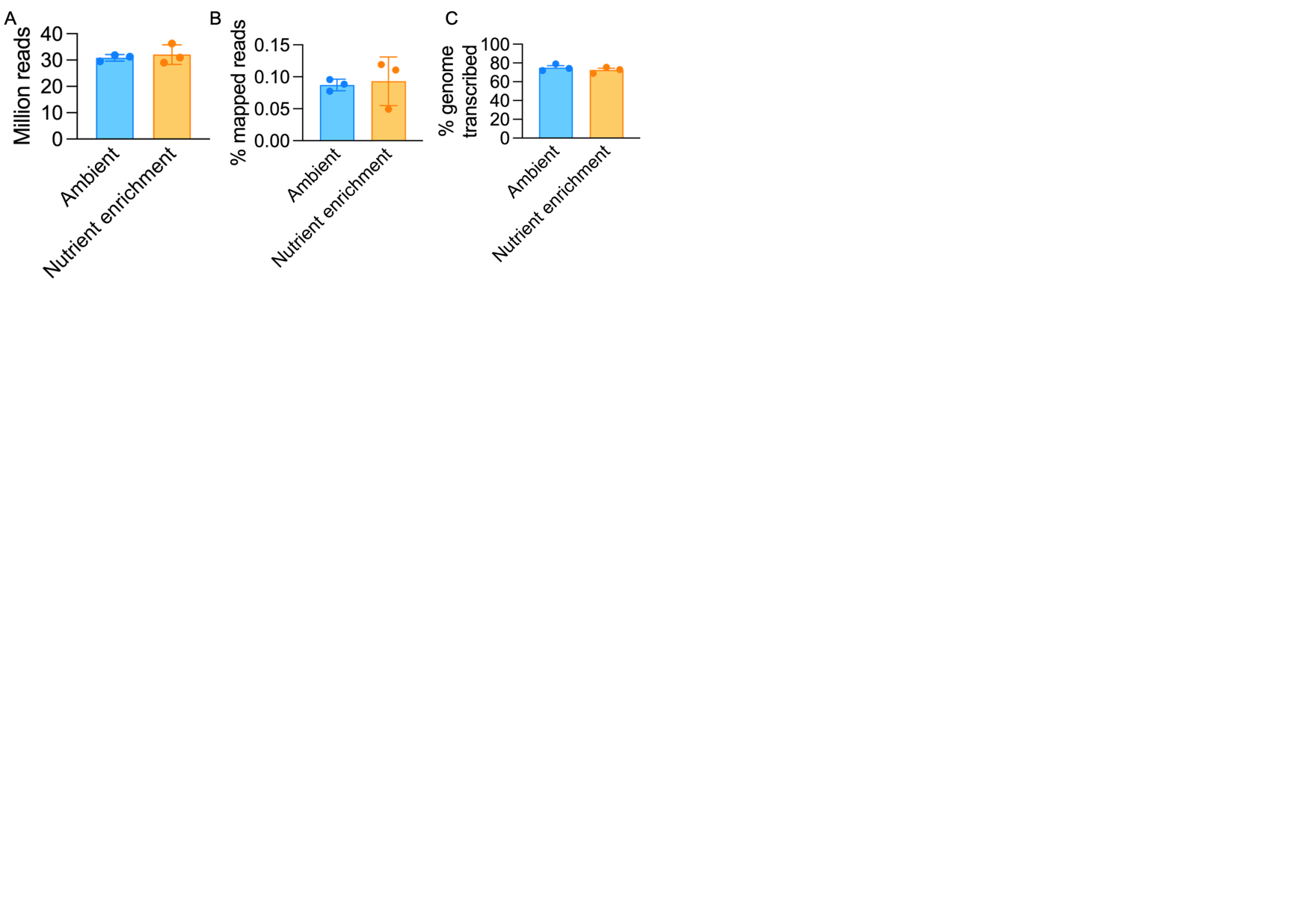


**Figure S1. Sequencing statistics for *Ca.* A. rohweri transcriptome.** Bar charts displaying (A) the number of reads for each sample after removing for low quality reads, (B) the percentage of reads that mapped to the *Ca.* A. rohweri transcriptome, and (C) the percentage of genes in the *Ca.* A. rohweri transcriptome that had at least one read. Values for each nutrient treatment were not statistically different from any other treatment in any of the panels shown above (One-way Analysis of Variance (ANOVA) with Tukey’s multiple comparison test, *P*>0.05).


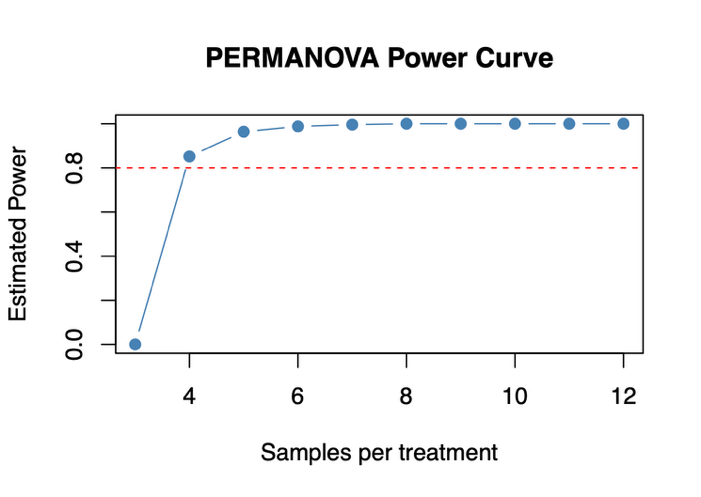


**Figure S2. Power Simulation analysis for transcriptomic experimental design.** Power was estimated across a range of sample and effect sizes to determine the sensitivity of the study to detect biologically meaningful differences. Curves represent the probability of detecting a true effect (a=0.05).

**
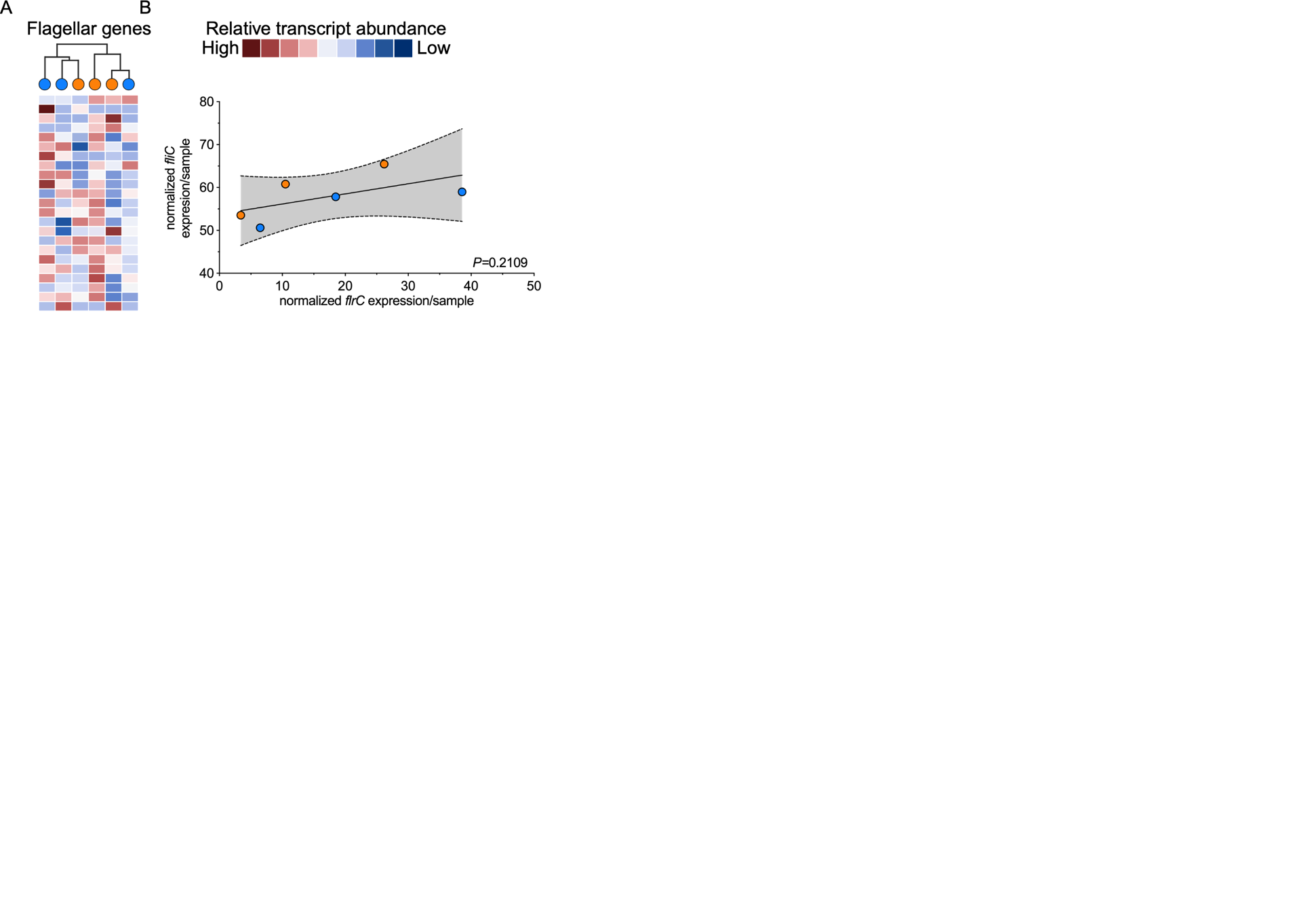
**

**Figure S3. Flagellar gene expression heatmap and correlation analysis.** (A) Heatmap displays relative transcript abundance for flagellar associated genes. Each circle within the hierarchical clustering analysis represents a sample and circle color indicates the experimental treatment: ambient (blue), and nutrient enrichment (orange). Relative transcript abundance is scaled across samples for a given gene where genes with relatively highly transcript abundance are shown in red and those with relatively low abundance are shown in blue. (B) Linear regression analysis between normalized *flrC* and *fliC* expression. Gray area indicates 95% confidence intervals.


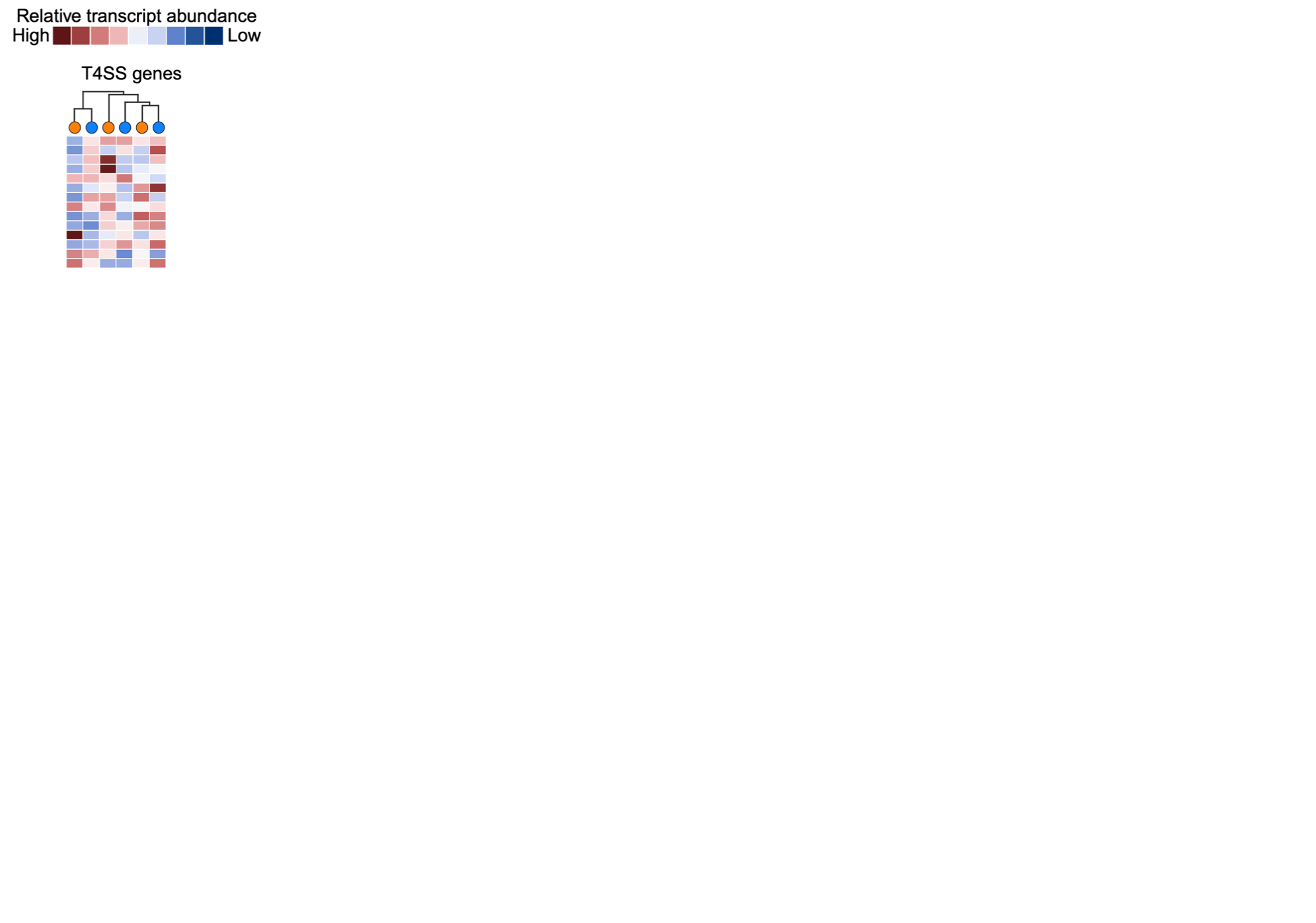


**Figure S4. Heatmap and hierarchical clustering of T4SS genes.** Heatmap displays relative transcript abundance for T4SS associated genes. Each circle within the hierarchical clustering analysis represents a sample and circle color indicates the experimental treatment: ambient (blue), and nutrient enrichment (orange). Relative transcript abundance is scaled across samples for a given gene where genes with relatively highly transcript abundance are shown in red and those with relatively low abundance are shown in blue.

**Figure S5. *Ca.* A. rohweri two component systems are phylogenetically congruent to one another.** Consensus phylogenetic trees constructed using two concatenated genes (ordered histidine kinase, response regulator) of the three two component systems found in *Ca.* A. rohweri (bolded). Node values were calculated by maximum likelihood (ML).

**Figure S6. *Ca.* A. rohweri two component systems are congruent to strain phylogeny.** Consensus phylogenetic tree constructed using six two component system genes (*ntrY, ntrX, envZ, ompR, phoR, phoB*), and 16S rRNA gene sequence (V4-V5) phylogenetic tree of 10 Rickettsiales strains. Node values were calculated by maximum likelihood (ML).
